# Last Words about the Epizooty

**Published:** 1873-10

**Authors:** 


### LAST WORDS ABOUT THE EPIZOOTY.

These last words are in the form of horse doctors' bills. We have seen several, but the following from a St. Louis veterinarian is the most striking.—

Sant Lewis Ganewerry the 4d 1873

Mr.———to james HanKox

Vetturerinary physickian and Surgeant Dr

| | |
|---|---|
| Too medikle advice twict | $ 3 00 |
| Konsultation over a ded mare sed too hev hed the ippyzout | 75 |
| Goin to see two sick hosses in the nite (very cold) | 2 00 |
| To treatment of a kream kollered hoss two days with medisuns | 4 50 |
| To making an obstetrikul examinashun of a hosses throat | 1 50 |
| To settin up all nite in a barn with a sick hoss | 2 50 |
| To writin a preeskripshun for botts, & also one for spaving | 1 00 |
| To holding a postmortim examinashun on a hoss who afterwards recovered | 1 50 |
| To givin my opinyun one day on the street regardin the kause of the zoot | 4 00 |
| Totil | $20 75 |

The gentleman who received the above will not contest the same on account of the charges, but will pay it cheerfully as soon as he receives a remittance from his parents.—*Med. & Sur. Reporter.*

